# Impact of blunt chest trauma on outcome after traumatic brain injury– a matched-pair analysis of the TraumaRegister DGU®

**DOI:** 10.1186/s13049-020-0708-1

**Published:** 2020-03-12

**Authors:** Mark Schieren, Frank Wappler, Arasch Wafaisade, Rolf Lefering, Samir G. Sakka, Jost Kaufmann, Hi-Jae Heiroth, Jerome Defosse, Andreas B. Böhmer

**Affiliations:** 1 Department of Anaesthesiology and Intensive Care Medicine, University Witten/Herdecke, Medical Center Cologne-Merheim, Ostmerheimer Str, 200, 51109 Cologne, Germany; 2 Department of Traumatology and Orthopedic Surgery, University Witten/Herdecke, Medical Center Cologne-Merheim, Cologne, Germany; 3 IFOM - Institute for Research in Operative Medicine, University Witten/Herdecke, Cologne, Germany; 4 Department of Intensive Care Medicine, University of Mainz, Gemeinschaftsklinikum Mittelrhein, Koblenz, Germany; 5 Department of Pediatric Anaesthesiology, University Witten/Herdecke, Children Hospital Amsterdamer Straße, Cologne, Germany; 6 Department of Neurosurgery, University Witten/Herdecke, Medical Center Cologne-Merheim, Cologne, Germany

**Keywords:** Traumatic brain injury, Thoracic injury, Glasgow Outcome Scale, Critical care, Registry

## Abstract

**Background:**

Traumatic brain injury (TBI) is associated with high rates of long-term disability and mortality. Our aim was to investigate the effects of thoracic trauma on the in-hospital course and outcome of patients with TBI.

**Methods:**

We performed a matched pair analysis of the multicenter trauma database TraumaRegisterDGU® (TR-DGU) in the 5-year period from 2012 to 2016. We included adult patients (≥18 years of age) with moderate to severe TBI (abbreviated injury scale (AIS)= 3–5). Patients with isolated TBI (group 1) were compared to patients with TBI and varying degrees of additional blunt thoracic trauma (AIS_Thorax_= 2–5) (group 2). Matching criteria were gender, age, severity of TBI, initial GCS and presence/absence of shock. The χ^2^-test was used for comparing categorical variables and the Mann-Whitney-U-test was chosen for continuous parameters. Statistical significance was defined by a *p*-value < 0.05.

**Results:**

A total of 5414 matched pairs (10,828 patients) were included. The presence of additional thoracic injuries in patients with TBI was associated with a longer duration of mechanical ventilation and a prolonged ICU and hospital length of stay. Additional thoracic trauma was also associated with higher mortality rates. These effects were most pronounced in thoracic AIS subgroups 4 and 5. Additional thoracic trauma, regardless of its severity (AIS_Thorax_ ≥2) was associated with significantly decreased rates of good neurologic recovery (GOS = 5) after TBI.

**Conclusions:**

Chest trauma in general, regardless of its initial severity (AIS_Thorax_= 2–5), is associated with decreased chance of good neurologic recovery after TBI. Affected patients should be considered “at risk” and vigilance for the maintenance of optimal neuro-protective measures should be high.

## Background

Traumatic brain injury (TBI) is associated with high rates of long-term disability and mortality. Main interventions in critical care, such as early tracheal intubation, mechanical ventilation, intracranial pressure monitoring - in order to quantify cerebral perfusion pressure - aim to reduce the risk for secondary neurologic injuries. Avoiding hypoxemia, hypercarbia and arterial hypotension are essential to maintain adequate tissue oxygenation and intracranial pressure (ICP) homeostasis. Presumably, the integrity of the respiratory system is a substantial element for optimal respiratory care in patients with moderate to severe TBI. In thoracic trauma, however, impaired alveolar gas exchange occurs frequently, resulting from lung contusions, pleural pathologies, disturbed ventilation mechanics or the development of acute respiratory distress syndrome (ARDS). A recently published retrospective analysis found severe thoracic trauma to be a risk factor for early-onset ventilator-associated pneumonia with negative consequences on cerebral oxygenation [1].

In addition, established supportive measures in respiratory care, such as lung protective ventilation, recruitment maneuvers, ventilation with high positive end-expiratory pressure (PEEP) levels, permissive hypercapnia, prone positioning or extracorporeal support may have detrimental effects on TBI [2]. Hence, we hypothesized that additional thoracic injuries may have a negative impact on outcome after traumatic brain injury. Therefore, the German trauma registry, TraumaRegister DGU®, was examined to compare clinical outcomes of patients with isolated TBI to those with comparable intracranial injury severity and baseline characteristics, yet with additional thoracic injuries of varying degrees.

## Methods

### Database

The multicenter TraumaRegister DGU® (TR-DGU) database was accessed to identify cases of TBI with and without additional blunt chest trauma to investigate its impact on neurologic and overall outcome. The TraumaRegister DGU® of the German Trauma Society was founded in 1993. The aim of this multi-center database is a pseudonymised and standardized documentation of severely injured patients. Data are collected prospectively in consecutive time phases from the site of the accident until discharge from hospital. The documentation includes detailed information on demographics, injury pattern, comorbidities, pre- and in-hospital management, course on intensive care unit and outcome of each individual. Trauma patients who are admitted via the emergency room with subsequent intensive care or intermediate care treatment (ICU/ICM) or those who reach the hospital with vital signs and die before admission to ICU are included. Participating hospitals are primarily located in Germany (90%), but a rising number of hospitals of other countries - with differing treatment algorithms - contribute data as well. Currently, approx. 35,000 cases from almost 700 hospitals are entered into the database per year. Participation in TraumaRegister DGU® is voluntary. For hospitals associated with TraumaNetzwerk DGU®, however, the entry of at least a basic data set is obligatory for reasons of quality assurance. As a compulsory tool for quality assessment, no informed consent is necessary for data collection. Participation in TraumaRegister-DGU® and analysis of data are approved by the participants’ institutional ethical review boards. Scientific data analysis is approved according to a peer-review procedure established by the Committee on Emergency Medicine, Intensive Care and Trauma Management (Sektion NIS) of the German Trauma Society. The TraumaRegister DGU® is approved by the review board of the German Trauma Society and is in compliance with the institutional requirements of its members. The present study is in line with the publication guidelines of the TraumaRegister DGU® and registered as TR-DGU project ID 2017–040.

### Patients

**Fig. 1:**
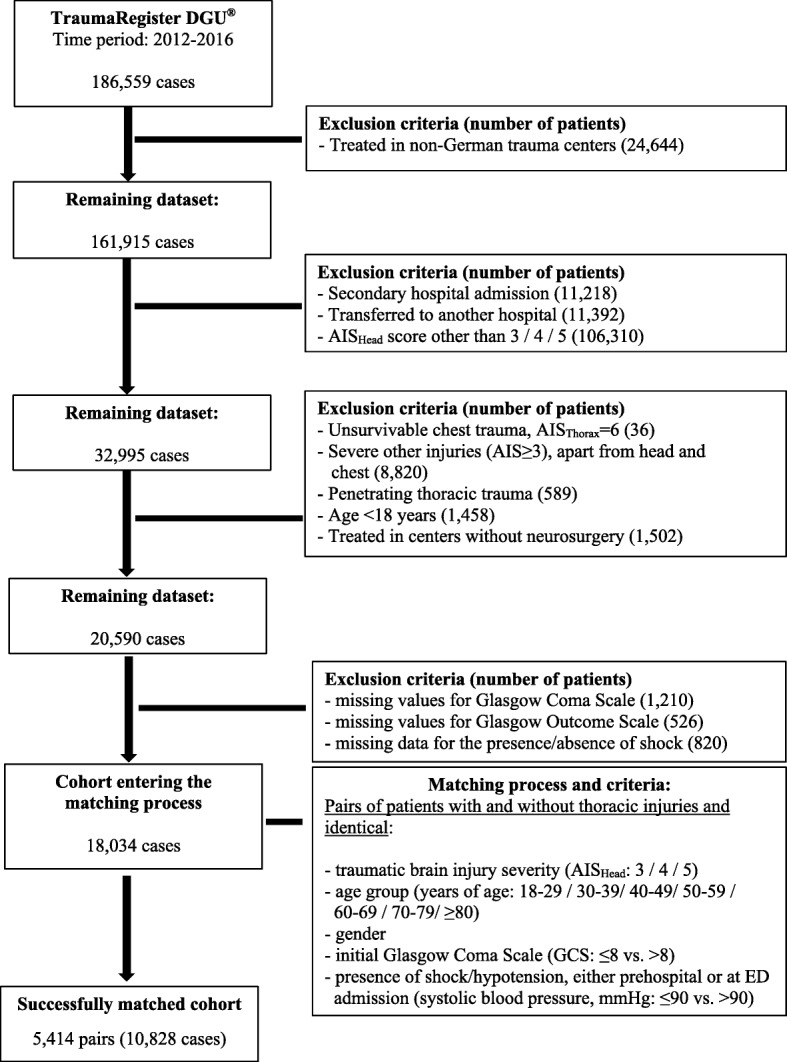
Flow chart outlining case selection and matching process

Patients in the 5-year period from 2012 to 2016 with complete basic data sets were screened. During that period 189,559 cases were entered into the database. We included adult patients (≥18 years of age) with TBI (abbreviated injury scale (AIS)_Head_ = 3, 4 or 5) who were primarily admitted to participating trauma centers in Germany (Fig. 1). The AIS_Head_ did not include injuries of the face and neck. Patients who were admitted secondarily or transferred to other hospitals as well as patients with severe injuries (AIS ≥3) in other body regions, apart from the head and chest, were excluded. Patients with penetrating chest trauma or unsurvivable/fatal thoracic injuries (AIS_Thorax_ = 6) were also excluded. Furthermore, we excluded cases that were treated in trauma centers without a department for neurosurgery or had missing values for the Glasgow Coma Scale (GCS), Glasgow Outcome Scale (GOS) or the presence/absence of shock, defined as systolic blood pressure ≤90 mmHg.

After applying inclusion and exclusion criteria, 18,034 patients entered the matching process. We identified matching pairs that had comparable gender, age, severity of traumatic brain injury, initial GCS, presence/absence of shock. To create two study groups, one partner of every pair had additional thoracic injuries of varying degrees (i.e. AIS_Thorax_ = 2, 3, 4 or 5) (Fig. 1).

Matching pairs were included in the statistical analysis to determine the impact of thoracic trauma on TBI outcome. The functional outcome after TBI, as described by the GOS at hospital discharge was the primary outcome of our study. Unfavorable outcomes were defined as in-hospital death (GOS = 1), persistent vegetative state (GOS = 2), severe disability (GOS = 3) or moderate disability (GOS = 4). The sample size of the study was determined by the availability of suitable matching pairs. We described basic patient characteristics and injury patterns and analyzed the hospital course as secondary outcomes.

### Statistical analysis

Statistical analysis was performed with SPSS Statistics® (IBM Corp., Armonk, USA, version 25). The χ^2^-test was used for comparing categorical variables and the Mann-Whitney-U-test was chosen for continuous parameters. Statistical significance was defined by a *p*-value < 0.05.

## Results

### Patients

A total of 5414 matched pairs (10,828 patients) was included (group 1 = TBI only; group 2 = TBI + chest trauma). There were no missing data. Both groups were comparable with regard to age, gender, and head trauma severity as well as rates of shock before/at emergency department (ED) admission and initial GCS ≤ 8 (Table 1). Car and motorbike accidents were more frequently seen in patients with chest trauma (Table 1). Patients with isolated TBI sustained their injuries most frequently from falls from low height (< 3 m). Patients in group 2 had additional chest trauma, resulting in higher values for injury severity scores (Table 1). The study cohort was divided into 4 subgroups according to chest trauma severity (AIS_Thorax_ = 2/3/4/5). Mean initial GCS values were comparable in all matched subgroups. There was a relevant proportion of the cases that either presented with very low or very high GCS scores (GCS score = 3: 21%; GCS score = 14/15: 34%). Moderate concomitant thoracic injuries (AIS_Thorax_ = 2–3), did not affect hemoglobin concentration, base excess, platelet counts, International Normalized Ratio (INR) and activated Partial Thromboplastin Time on hospital admission (Additional file 1: Table S1). In case of more severe thoracic trauma (AIS_Thorax_ = 4–5), however, admission laboratory values demonstrate lower hemoglobin concentration and base excess and an increased activated Partial Thromboplastin Time (Additional file 1: Table S1).

**Table 1:** Basic characteristics of the matched study groups (*n* = 10,828 patients)

Patient characteristics	Group 1 _TBI only_	Group 2 _TBI + Chest Trauma_
Number of patients	5414	5414
Results presented as means (SD)		
Age (years)^a^	56.2 (20.5)	56.1 (20.6)
ISS	17.3 (6.5)	27.8 (8.6)
Results presented as number of patients (% per group)		
Male gender^a^	4026 (84.4)	4026 (84.4)
Shock before/at ED admission^a^	742 (13.7)	742 (13.7)
Initial GCS ≤ 8^a^	2237 (41.3)	2237 (41.3)
AIS_Head_ = 3^a^	2317 (42.8)	2317 (42.8)
AIS_Head_ = 4^a^	1730 (32.0)	1730 (32.0)
AIS_Head_ = 5^a^	1367 (25.2)	1367 (25.2)
AIS_Thorax_ = 2	none	1222 (22.6)
AIS_Thorax_ = 3	none	3076 (56.8)
AIS_Thorax_ = 4	none	772 (14.3)
AIS_Thorax_ = 5	none	344 (6.4)
Mechanism of injury		
- MVA, car occupant	450 (8.3)	1001 (18.5)
- MVA, motor bike occupant	210 (3.9)	490 (9.1)
- MVA, bicycle occupant	706 (13.0)	736 (13.6)
- MVA, pedestrian	312 (5.8)	417 (7.7)
- Fall > 3 m	647 (12.0)	1057 (19.5)
- Fall ≤ 3 m	2289 (42.3)	1310 (24.2)
- Other	638 (11.8)	321 (5.9)
- Unknown	162 (3.0)	82 (1.5)

^a^ matching criteria applying to both study groups

*GCS* Glasgow Coma Scale, *SD* Standard deviation

*ISS* Injury Severity Score, *AIS* Abbreviated Injury Scale

*ED* Emergency department, *TBI* Traumatic brain injury

*MVA* Motor vehicle accident

### Impact of chest trauma on ICU and hospital course after TBI

In patients with TBI, the presence of additional thoracic injuries was associated with a significantly longer duration of mechanical ventilation and prolonged ICU and hospital length of stay (Table 2). In case of additional thoracic injuries (AIS_Thorax_ = 3–5), the need for red blood cell transfusion within the first 48 h after hospital admission was higher. The presence of chest injuries was also associated with higher overall mortality rates after TBI (Table 2).

**Table 2:** Clinical outcomes of the matched study groups (*n* = 10.828 patients)

Outcome variable	Group 1 _TBI only_	Group 2 _TBI + Chest trauma_	*p*
Number of patients per group	5414	5414	
Results presented as mean / median (SD)			
Duration of mechanical ventilation (days)	4.2 / 1 (8.2)	5.9 / 1 (9.4)	**< 0.001**
ICU length of stay (days)	7.7 / 3 (10.7)	10.1 / 5 (11.7)	**< 0.001**
Hospital length of stay (days)	14.8 / 11 (14.4)	17.7 / 14 (15.1)	**< 0.001**
Results presented as number of patients (% per group)			
PRBC transfusion required	172 (3.2)	351 (6.5)	**< 0.001**
Neurological outcome of survivors (Glasgow Outcome Scale)			
Death (GOS = 1)	1077 (19.9)	1186 (21.9)	**0.010**
Persistent vegetative state (GOS = 2)	159 (2.9)	193 (3.6)	0.066
Severe disability (GOS = 3)	538 (9.9)	634 (11.7)	**0.003**
Moderate disability (GOS = 4)	1096 (20.2)	1233 (22.8)	**0.001**
Good recovery (GOS = 5)	2544 (47.0)	2168 (40.0)	**< 0.001**

*PRBC* Packed red blood cells, *ED* Emergency department

*AIS* Abbreviated Injury Scale, *GOS* Glasgow Outcome Scale

### Impact of chest trauma on neurologic recovery after TBI

**Fig. 2:**
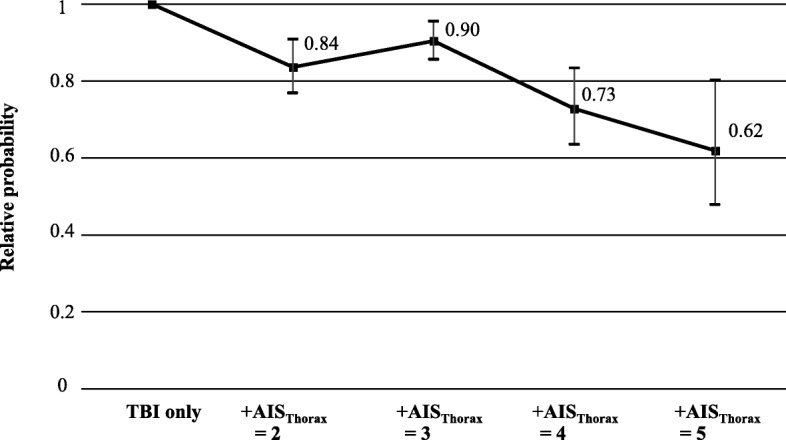
Impact of additional chest trauma (AIS_Thorax_ = 2 / 3 / 4 / 5) on relative probability of good neurologic recovery (GOS = 5) after TBI. Each AIS_Thorax_-subgroup was compared to their respective matching partners with identical TBI severity and baseline characteristics, yet without additional thoracic trauma. Error bars indicate 95%-confidence intervals of the relative probability

Additional thoracic trauma was associated with worse neurologic outcome after TBI (Table 2 and Fig. 2). Unfavorable outcomes (GOS = 1–4) were more likely and good neurologic recovery (GOS = 5) occurred less frequently compared to patients with isolated TBI (Table 2). The negative impact of thoracic trauma on outcomes was most pronounced with regard to the number of patients that were discharged with good neurologic function. The chance of good neurologic recovery decreased if patients sustained additional thoracic injuries, regardless of the severity of these injuries (i.e. AIS_Thorax_ ≥ 2) (Fig. 2).

## Discussion

This matched pair analysis investigating the impact of chest trauma on outcome after TBI, demonstrated that additional thoracic injuries are associated with higher mortality rates as well as longer duration of mechanical ventilation, ICU and hospital length of stay, confirming previous findings from smaller cohorts [3, 4]. However, it is a novel finding that chest trauma in general, regardless of its initial severity (i.e. AIS_Thorax_ ≥ 2), is associated with decreased chance of good neurologic recovery after TBI. Impaired neurologic recovery would be expected in case of serious or critical thoracic injuries, as these are frequently associated with severe hypoxemia or hypercarbia (i.e. pleural pathologies, lung contusions, ARDS) and circulatory shock - conditions which are known to have subsequent detrimental effects on TBI outcome (i.e. hypoxemia, increased ICP, poor cerebral perfusion) [4–11]. However, according to our results even minor forms of chest trauma (i.e. AIS = 2) are likely to decrease chance of good neurologic recovery, although one would expect that gas exchange and systemic perfusion would be less impaired by minor thoracic injuries.

So far, evidence on the impact of chest trauma on functional outcome after TBI is mostly limited to small retrospective analyses. Results of a previous unmatched retrospective single center analysis of 505 TBI patients indicate that a Pulmonary Contusion Score ≥ 6, AIS_Thorax_ ≥ 3, increasing age, increasing Thoracic Trauma Severity Score values, lower GCS, and abnormal pupillary function at hospital admission were associated with higher rates of unfavorable outcomes (i.e. GOS = 1–3) [3]. Given the unmatched nature of the study, especially with regard to initial GCS and low incidence of relevant chest trauma (AIS ≥ 3 = 8.1% of patients), the external validity of these findings and comparability to our study is limited. Our results are in line with those from a paediatric cohort that also found decreased rates of favorable neurologic function at hospital discharge and 6 months after discharge in case patients with TBI presented with pulmonary contusions [4].

However, there is also a study that could not demonstrate a negative impact of chest trauma on outcome after TBI. This retrospective matched pair analysis of polytraumatized patients with TBI did not find a negative impact of concomitant pulmonary contusions and impaired paO_2_/FiO_2_-ratio on morbidity or mortality [8]. Considering that other conditions harmful to neurologic recovery, such as arterial hypotension, coagulopathy, hypovolemia and anemia are common in patients with multiple trauma, the additional impact of impaired paO_2_/FiO_2_-ratio on overall outcome may be limited. However, the study may have been underpowered (*n* = 180) to detect statistically significant differences.

Our findings should raise awareness regarding the identification of patients at risk and maintenance of optimal neuro-supportive conditions after TBI. To allow for adequate risk stratification of patients with TBI presenting with a relevant mechanism of injury, yet without clinical signs of thoracic injuries at hospital admission, a chest CT scan may be warranted to assess the presence and severity of additional undetected thoracic injuries. This is of particular importance in elderly patients, who are at an increased risk for TBI. In the elderly, TBI is most commonly sustained from low energy mechanisms, such as falls [12, 13]. In this context, however, CT imaging of the chest may not necessarily be part of the workup of every patient with presumed isolated TBI. However, thoracic trauma is common in the elderly and minor injuries may be missed without CT, as they may present with normal clinical exam and chest x-ray findings [14, 15]. Without the recognition of the harmful effects of concomitant minor chest trauma, risk stratification and appropriate patient disposition is not possible. Furthermore, secondary hits on cerebral metabolism, such as hypoxemia, hypercarbia, significant anemia, poor cerebral perfusion must be avoided. In non-intubated patients, early use of non-invasive ventilation or high-flow nasal oxygen may provide suitable treatment options to improve pulmonary function and gas exchange. Adequate analgesia is of particular importance in thoracic trauma to improve respiratory mechanics and autonomous mobilisation of bronchial secretions. Early treatment (e.g. chest drain) of posttraumatic pleural pathologies may be helpful to decrease the risk for respiratory impairment. Furthermore, maintenance of adequate cerebral perfusion (i.e. early vasopressor therapy and volume replacement) and normoglycemia are helpful to avoid secondary neurologic insults. This may be difficult in patients with hemorrhage from additional injuries, as blood pressure levels required for adequate cerebral perfusion may increase blood loss from uncontrolled bleeding sites. As even brief episodes of hypotension may have significant impact on TBI mortality and functional outcome, the risk and benefits of adequate cerebral perfusion must be carefully weighed [9].

As any retrospective analysis of registry data, our study is subject to certain limitations. As we are restricted to the recorded items of the registry data set, not all information of interest (e.g. incidence/severity of hypoxemia or hypercarbia, cerebral perfusion and ICP values) is available. Although we are able to demonstrate that chest trauma is associated with worse outcome after TBI, we can merely speculate on causative mechanisms. In case of severe chest trauma (AIS_Thorax_ = 4–5), laboratory values at hospital admission and transfusion requirements may indicate higher rates of hemorrhage, coagulopathy and shock. This would certainly be expected to negatively influence TBI outcome. For less severe thoracic injuries (AIS_Thorax_ = 2–3), however, we could not deduce a specific potential causative mechanism from the available data. Chest trauma is related to several determinants of TBI outcome, yet, based on the dataset of the TraumaRegister DGU® further differentiation of particular effects is not possible. Apart from compromising respiratory function, the presence of chest trauma inevitably increases overall injury severity and thereby contributes to posttraumatic systemic inflammatory response, which is known to exert negative effects on outcome after TBI [16]. However, as details on ventilation strategies, gas exchange or inflammatory markers are missing from the registry’s dataset, the extent and interaction of these effects remain unclear. The evaluation of the neurological function of survivors by the GOS is limited to 4 major categories. As the spectrum of disability within the categories is relatively broad and the classification of patient status may be biased by subjective interpretation of treating physicians, a more objective and detailed assessment of neurologic function at hospital discharge would be desirable. Good neurologic recovery was defined as a GOS score of 5. Compared to other GOS scores (i.e. GOS 2–4), we considered a GOS of 5 to be a relatively unambiguous description of neurologic function. Furthermore, optimizing the chance for good neurologic recovery is the main goal of TBI therapy.

The external validity of our findings are limited to adult patients with TBI that are treated in trauma and critical care systems similar to German standards of care.

As the TraumaRegister DGU® observation period ends at hospital discharge, the effect of thoracic trauma on long-term outcome after TBI remains unclear. Although the long-term predictive accuracy of GOS levels after TBI has been demonstrated [17], it is unclear whether there are differences in delayed neurologic recovery, which occurs in up to 30% of patients [18].

Future analyses that closely analyze vital signs (e.g. ICP and cerebral perfusion pressure) and gas exchange, specific posttraumatic inflammatory, pulmonary, circulatory or intracranial complications as well as long term outcome are required for a differentiated assessment of the negative impact of chest trauma on neurologic function.

## Conclusion

We conclude that additional chest trauma, regardless of its severity, is associated with worse outcome after TBI. There is urgent need for investigation of causative mechanisms. Furthermore, affected patients should be considered “at risk” and vigilance for the maintenance of optimal neuro-protective measures should be high.

## Supplementary information

**Additional file 1: Table S1.** Laboratory results at the time of hospital admission of the matched study groups (*n* = 10,828 patients).

## Data Availability

Apart from data included in the manuscript supporting the conclusions of this article, datasets of the TraumaRegisterDGU® are not publicly accessible. Data access requires permission by the Committee on Emergency Medicine, Intensive Care and Trauma Management (Sektion NIS) of the German Trauma Society.

## Abbreviations

AIS: Abbreviated injury scale
ARDS: Acute respiratory distress syndrome
ED: Emergency department
GCS: Glasgow Coma Scale
GOS: Glasgow Outcome Scale
ICP: Intracranial pressure
ICU: Intensive care unit
INR: International Normalized Ratio
PEEP: Positive end-expiratory pressure
PRBC: Packed red blood cells
SIRS: Systemic inflammatory response syndrome
TBI: Traumatic brain injury
TR-DGU: TraumaRegisterDGU®
