# Supplementary material for: Impact of blunt chest trauma on outcome after traumatic brain injury– a matched-pair analysis of the TraumaRegister DGU®
Source: Scand J Trauma Resusc Emerg Med. 2020 Mar 12;28:21. doi: 10.1186/s13049-020-0708-1 (PMC7069167; doi:10.1186/s13049-020-0708-1)
Supplement: Supplementary file 1 — Additional file 1: Table S1. Laboratory results at the time of hospital admission of the matched study groups (n = 10,828 patients). [file 13049_2020_708_MOESM1_ESM.docx]

Additional file 1: **Table S1.** Laboratory results at the time of hospital admission of the matched study groups (n=10,828 patients).

| Laboratory findings | Group 1 _TBI only_ | Group 2 _TBI + chest trauma_ | |
| --- | --- | --- | --- |
| Base excess in mmol/l | |  |  |
|  | -1.5 (3.9) | + AIS_Thorax_ = 2 | -1.8 (4.2) |
|  | -1.8 (4.4) | + AIS_Thorax_ = 3 | -1.8 (4.5) |
|  | -2.5 (5.2) | + AIS_Thorax_ = 4 | -2.8 (5.0) |
|  | -2.8 (5.6) | + AIS_Thorax_ = 5 | -4.7 (6.6) |
|  | | | |
| Hemoglobin concentration in g/dl | | | |
|  | 13.5 (1.9) | + AIS_Thorax_ = 2 | 13.2 (2.0) |
|  | 13.4 (2.0) | + AIS_Thorax_ = 3 | 13.1 (2.0) |
|  | 13.2 (2.2) | + AIS_Thorax_ = 4 | 12.6 (2.4) |
|  | 13.1 (2.2) | + AIS_Thorax_ = 5 | 12.3 (2.5) |
|  |  |  |  |
| Activated prothrombin time in seconds | | | |
|  | 29.5 (10.8) | + AIS_Thorax_ = 2 | 30.3 (11.8) |
|  | 30.1 (12.6) | + AIS_Thorax_ = 3 | 29.9 (12.0) |
|  | 31.2 (14.9) | + AIS_Thorax_ = 4 | 34.9 (22.7) |
|  | 33.1 (18.4) | + AIS_Thorax_ = 5 | 36.9 (28.2) |
| International normalized ratio | | |  |
|  | 1.2 (0.6) | + AIS_Thorax_ = 2 | 1.2 (0.5) |
|  | 1.2 (0.6) | + AIS_Thorax_ = 3 | 1.2 (0.5) |
|  | 1.2 (0.5) | + AIS_Thorax_ = 4 | 1.3 (0.8) |
|  | 1.3 (1.1) | + AIS_Thorax_ = 5 | 1.4 (0.6) |
|  |  |  |  |
| Platelet count (x 10^9^/l) | | |  |
|  | 222 (82) | + AIS_Thorax_ = 2 | 220 (71) |
|  | 213 (82) | + AIS_Thorax_ = 3 | 210 (78) |
|  | 207 (78) | + AIS_Thorax_ = 4 | 208 (75) |
|  | 202 (78) | + AIS_Thorax_ = 5 | 202 (70) |

*AIS = Abbreviated Injury Scale SD= standard deviation TBI= traumatic brain injury*

Each AIS_Thorax_-subgroup was compared to their respective matching partners with identical TBI severity, yet without additional thoracic trauma. Results presented as means (SD).
